# Identification of Reference Genes for Relative Quantification of Circulating MicroRNAs in Bovine Serum

**DOI:** 10.1371/journal.pone.0122554

**Published:** 2015-03-31

**Authors:** In-Seon Bae, Ki Yong Chung, Jongmin Yi, Tae Il Kim, Hwa-Sik Choi, Young-Moo Cho, Inho Choi, Sang Hoon Kim

**Affiliations:** 1 Department of Biology, Kyung Hee University, Seoul, Republic of Korea; 2 National Institute of Animal Science, RDA, Pyeongchang, Gangwon, Republic of Korea; 3 Department of Biomedical Laboratory Science, Shinhan University, Uijeongbu, Gyeonggi, Republic of Korea; 4 Bovine Genome Resources Bank, School of Biotechnology, Yeungnam University, Gyeongsan, Gyeongbuk, Republic of Korea; University of Catanzaro Magna Graecia, ITALY

## Abstract

Circulating microRNAs in body fluids have been implicated as promising biomarkers for physiopathology disorders. Currently, the expression levels of circulating microRNAs are estimated by reverse transcription quantitative real-time polymerase chain reaction. Use of appropriate reference microRNAs for normalization is critical for accurate microRNA expression analysis. However, no study has systematically investigated reference genes for evaluating circulating microRNA expression in cattle. In this study, we describe the identification and characterization of appropriate reference microRNAs for use in the normalization of circulating microRNA levels in bovine serum. We evaluated the expression stability of ten candidate reference genes in bovine serum by using reverse transcription quantitative real-time polymerase chain reaction. Data were analyzed using geNorm, NormFinder, and BestKeeper statistical algorithms. The results consistently showed that a combination of miR-93 and miR-127 provided the most stably expressed reference. The suitability of these microRNAs was validated, and even when compared among different genders or breeds, the combination of miR-93 and miR-127 was ranked as the most stable microRNA reference. Therefore, we conclude that this combination is the optimal endogenous reference for reverse transcription quantitative real-time polymerase chain reaction-based detection of microRNAs in bovine serum. The data presented in this study are crucial to successful biomarker discovery and validation for the diagnosis of physiopathological conditions in cattle.

## Introduction

MicroRNAs (miRNAs) are small non-coding RNAs, which are widely expressed in the genomes of plants, animals, and humans [1]. Recent studies have demonstrated that serum and plasma contain large amounts of miRNAs, which are stabilized and protected from RNAse degradation by inclusion in various protein complexes, microvesicles, or exosomes [2–4]. Circulating miRNAs have the potential to serve as biomarkers for changes in physiological conditions such as pregnancy and in pathological conditions such as cancer, diabetes, and other diseases. Therefore, it is important to measure miRNA expression in the blood with high accuracy.

Quantitative real-time PCR (qRT-PCR) is currently the most frequently used approach for the evaluation of circulating miRNAs [5–7]. Due to its high sensitivity, specificity, good reproducibility, and cost-effectiveness, this is a powerful technique for measuring the expression profile of miRNAs. The accuracy of qRT-PCR-based miRNA expression analysis depends on an appropriate normalization by using reference genes. Thus, the optimal selection of genes to be used for normalization is critical for qRT-PCR data analysis.

In miRNA expression studies, the most commonly used reference genes are ribosomal RNAs such as 5S RNA and small nuclear RNAs like RNU6B. However, 5S RNA and RNU6B are degraded in some serum samples [8–10]. Endogenous miRNAs in solid tissues have been used as reference miRNAs. For instance, miR-23a and miR-191 are used for normalization in profiling studies of human cervical tissues [9], and let-7a and miR-16 are selected as reference genes in human breast cancer tissues [11]. However, in livestock, few studies have been described so far, with the exception of studies using porcine tissues where miR-93, miR-25, miR-106a, miR-17-5p, and miR-26a have been reported as stable reference miRNAs [12–13]. In the case of circulating miRNAs, endogenous normalizers have been well studied in humans and mice. For example, miR-146a, miR-16, miR-195, miR-30e, and miR-744 are stably expressed in mouse serum [4], and miR-16 is used as an internal normalizer in the serum of human B-cell lymphoma and colorectal cancer patients [14, 15]. In addition, the combination of let-7d, let-7g, and let-7i was recently reported as a normalizer of human serum miRNAs [16]. To date, no study has reported endogenous normalizers for circulating miRNAs in cattle. However, quantification of miRNA levels in bovine blood is essential for gaining further insight into their biological function and investigating potential biomarkers for bovine disease and traits of economic importance.

In the present study, we determined optimal reference miRNAs to be used for normalization of qRT-PCR data in bovine serum samples. We first identified invariant miRNAs as candidate reference miRNAs whose expression levels were stable in bovine serum. Appropriate reference miRNAs were identified by using geNorm, NormFinder, and BestKeeper statistical algorithms. In this study, we show that the combination of miR-93 and miR-127 serves as a stable reference in bovine serum for the normalization of circulating miRNAs.

## Materials and Methods

### Serum sample preparation

Blood samples of Korean native cattle (n = 33) and Holstein dairy cows (n = 16) were collected at National Institute of Animal Sciences, Suwon, Korea. All experimental involving live animals were approved by the Animal Care and Use Committee in National Institute of Animal Sciences of Rural Development Administration, Korea. For breed miRNA study, bovine serum was collected from steer (n = 14), bull (n = 9), and heifer (n = 10) of Korean native cattle. Samples were centrifuged at 5,000 rpm for 20 min at 4°C. The supernatant was removed and stored at −80°C until analysis. The degree of hemolysis in serum was analyzed by using spectrophotometry at 414 nm wavelength. Samples were classified to be hemolyzed when exceeded 0.2 value at 414 nm measurement.

### Extraction of miRNA, cDNA preparation, and qRT-PCR

Samples were thawed on ice, and miRNA was extracted using a serum miRNA purification kit (Genolution Inc., Seoul, Republic of Korea) by following the manufacturer’s instructions. The quantity and purity of extracted miRNA were estimated by monitoring both the absorbance at 260 nm and the 260/280 nm ratio. Next, 0.1 μg of RNA per sample was reverse-transcribed to cDNA with a miScript Reverse Transcription Kit (Qiagen, Hilden, Germany). This cDNA was then diluted 1:20 in water, after which 2 μl was used for quantitative PCR by using the miScript SYBR Green Kit (Qiagen, Hilden, Germany) with a Rotor-Gene Q PCR instrument (Qiagen, Hilden, Germany). Primers for mature reference miRNAs were obtained from Qiagen (Hilden, Germany). The reaction was performed at 94°C for 15 min, followed by 40 cycles of 94°C for 15 sec, 55°C for 30 sec, and 70°C for 20 sec. PCR efficiency (E) for each miRNA was determined with the slope of a linear regression model by using Cq values and the following equation: E = [10^(1/-slope)^−1] × 100%.

### Data analysis for miRNA stability

To assess the stability of candidate reference miRNAs, statistical algorithms including geNorm, NormFinder, and BestKeeper were utilized. The geNorm program is based on the mean pairwise variation for a given gene compared to the remaining tested genes, which are to calculate an M value as a gene expression stability factor [17–19]. The reference gene combination was calculated by geometric mean of their expression levels [17]. NormFinder is an ANOVA-based model to provide direct measure of intra- and inter-group variation in expression for each gene [20, 21]. BestKeeper program is used to determine the geometric mean and standard deviation of the Cq values of the candidate genes by pairwise correlation analyses [22].

### Statistical analysis

Statistical analyses were performed with SPSS 18 statistical software (SPSS Inc., Chicago, IL, USA). One-way ANOVA was used to determine differences in the relative expression levels of miRNAs among different groups.

## Results

### Expression levels of candidate reference miRNAs

For the evaluation of potential endogenous normalizers in bovine serum, ten candidate reference miRNAs were selected from other species and our preliminary next-generation sequencing. Nine miRNAs out of ten candidate reference miRNAs have been described in the literature as reference miRNAs in other species [4, 5, 8, 9, 11–13]. miR-127 was selected from our preliminary next-generation sequencing for the profiling of bovine skeletal muscle. The performance of each primer pair was tested by qRT-PCR. The amplification efficiencies for the ten primers ranged from 93.07% to 103.53%, according to the slopes of the standard curves (S1 Table). Melting curve analysis confirmed the presence of a single PCR product with a single peak from all samples, indicating that all the primers used are highly specific and efficient for qRT-PCR amplification.

**Fig 1:**
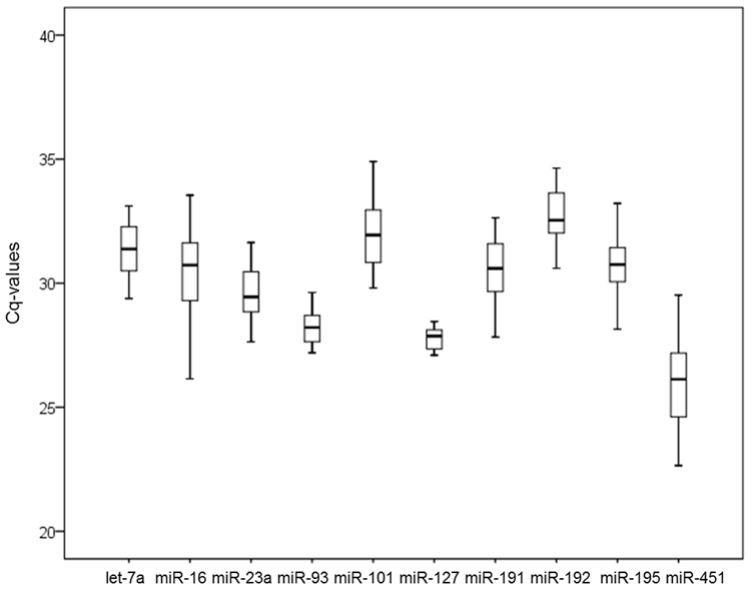
Expression levels of candidate reference miRNAs in bovine serum. The expression levels of ten candidate miRNAs were validated in bovine serum. Box plots of raw Cq values were produced. Boxes represent the lower and upper quartiles with median (n = 34). Values are given as real-time PCR cycle threshold numbers. Box and whisker plot displaying the range of Cq values for each putative reference miRNA. The median is marked by the middle line in the box. The boxes represent the 25th and 75th percentiles, and ranges are represented by the whiskers.

As shown in Fig 1, the quantification cycle (Cq) values of the ten candidate genes ranged from 24.06 to 36.92. The most highly expressed gene was miR-451, which exhibited a median Cq value of 25.62. All other genes had median Cq values larger than 27, and miR-192 presented the lowest level of expression with a median Cq value of 32.41. The range of Cq values indicates the stability of reference gene expression, and the wider the range of Cq values for the gene, the more unstable the gene’s expression. The range of Cq values for miR-16, miR-451, and miR-101 was above six cycles, range wider than that of the other genes tested. miR-101 was higher standard deviation than miR-16 and miR-451. The range of Cq values for miR-127 and miR-93 was 1.36 cycles and 2.43 cycles, respectively. The standard deviation for miR-127 and miR-93 was lowest among the genes tested, indicating that they are more stably expressed than other genes. Calculating the Cq value is important to select the endogenous reference standard. However, simple comparison of the raw Cq data for candidate reference miRNAs does not provide enough information. Therefore, we conducted additional analysis by using three different statistical algorithms for validation of reference miRNAs.

### Expression stability of candidate reference miRNAs

**Fig 2:**
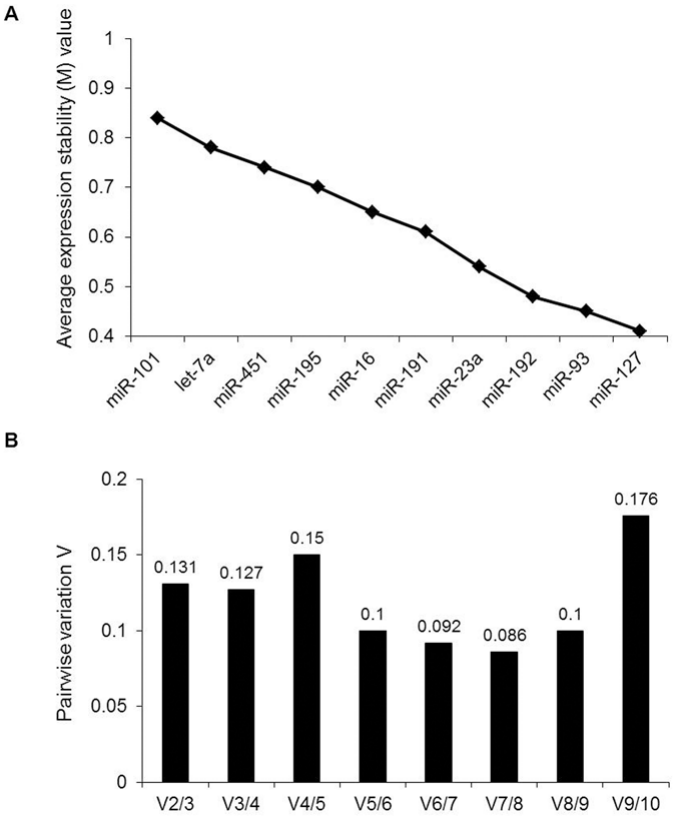
geNorm analysis of qRT-PCR of candidate reference genes. (A) Ranking of candidate reference genes according to average expression stability (M value). The x-axis from left to right indicates the ranking of the genes according to their expression stability. (B) Determination of the optimal number of reference genes for normalization. The software calculates the normalization factor from at least two genes; the variable V defines the pairwise variation between two sequential normalization factors.

Three distinct statistical algorithms, geNorm, NormFinder, and BestKeeper, were used to assess the stability of the candidate reference miRNAs. geNorm represents the average expression stability of candidate genes with M values. The larger the M value, the lower the stability. Fig 2A shows the results of geNorm analysis of all ten candidates. As shown, miR-127 (M = 0.41) and miR-93 (M = 0.45) were the most stable miRNAs, followed by miR-192, miR-23a, miR-191, miR-16, miR-195, miR-451, let-7a, and miR-101. geNorm also presents the optimal number of reference genes required for accurate normalization. For pairwise variation Vn/Vn+1, 0.15 is the proposed cut-off value. A Vn/Vn+1 value less than 0.15 implies that the top n reference genes are sufficient as internal controls. In our study, the V2/3 value was 0.131 (Fig 2B), suggesting that the top two reference miRNAs (miR-127 and miR-93) would be adequate for normalization, and the additional third miRNA is not necessary.

**Fig 3:**
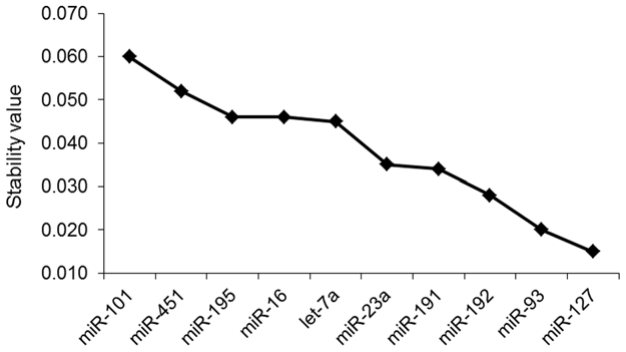
Stability values of each reference miRNA from the NormFinder algorithm. Ranking of candidate reference genes based on stability values calculated by NormFinder.

Subsequently, the expression data were analyzed with NormFinder. This program ranked the candidate miRNAs based on intra- and inter-group variations, with a lower stability value indicating more stable expression. As shown in Fig 3, NormFinder ranked the ten candidate miRNAs from lowest to highest stability value as follows: miR-127, miR-93, miR-192, miR-191, miR-23a, let-7a, miR-16, miR-195, miR-451, and miR-101. Like geNorm, NormFinder also identified miR-127 and miR-93 as the most stably expressed miRNAs in all samples, with miR-101 being the least stably expressed. Only small differences in the rankings calculated by the two programs were observed: miR-191 and let-7a were ranked fifth and ninth in geNorm, while they were ranked fourth and sixth in NormFinder, respectively.

BestKeeper analyzes gene expression variation for candidate genes by calculating standard deviation (SD) and a pairwise correlation. The lowest SD value indicates the most stable reference miRNA expression. As shown in Table 1, miR-127 and miR-93 were the most stably expressed genes with the lowest SDs (0.27 and 0.46, respectively). In this respect, the results obtained through the BestKeeper program were consistent with those obtained through the geNorm and NormFinder analyses. An overall ranking of candidate reference miRNAs was obtained and the recommended comprehensive rankings are given in Table 1. miR-127 and miR-93 represent the most adequate normalization candidate miRNAs tested in this study.

**Table 1:** Ranking of candidate miRNAs according to their stability value by using geNorm, NormFinder, and BestKeeper statistical algorithms.

Name	geNorm		NormFinder		BestKeeper		Consensus
	Stability value	Rank	Stability value	Rank	SD^1^	Rank	
let-7a	0.78	9	0.045	6	1.37	9	9
miR-16	0.65	6	0.046	7	1.27	8	6
miR-23a	0.54	4	0.035	5	0.94	4	4
miR-93	0.45	2	0.020	2	0.46	2	2
miR-101	0.84	10	0.060	10	1.62	10	10
miR-127	0.41	1	0.015	1	0.27	1	1
miR-191	0.61	5	0.034	4	1.11	6	5
miR-192	0.48	3	0.028	3	0.85	3	3
miR-195	0.7	7	0.046	8	1.08	5	7
miR-451	0.74	8	0.052	9	1.141	7	8

^1^SD, standard deviation

### Expression levels of candidate miRNAs in hemolyzed bovine serum

**Fig 4:**
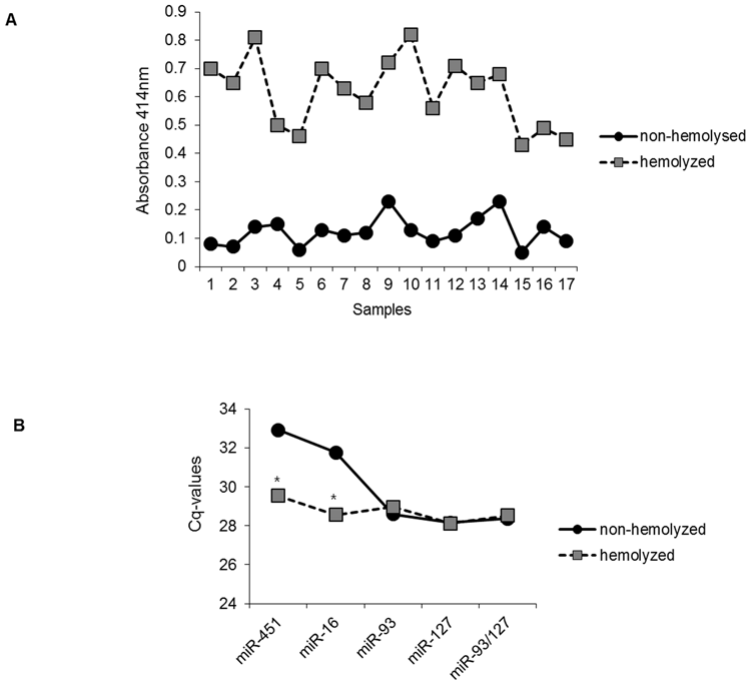
Evaluation of potential reference miRNAs in hemolyzed bovine serum. (A) The absorbance values at 414nm of hemolyzed and non-hemolyzed serum were measured to determine levels of free hemoglobin. (B) Levels of miR-16, miR-451, miR-93, miR-127, and the combination miR-93/miR-127 were measured in a cohort of non-hemolyzed samples (circles, n = 17) and hemolyzed samples (squares, n = 17). Statistical analysis was performed using Student’s *t*-test. *p < 0.05.

During the preparation of bovine serum from whole blood, some serum samples exhibited pink or red discoloration, apparently from hemolysis, which may have affected the level of miRNAs in the serum. In our study, we determined the expression levels of potential candidate reference miRNAs in hemolyzed serum compared to non-hemolyzed serum. Firstly, we observed whether bloods tested were hemolyzed by spectrophotometry. As shown in Fig 4A, hemolyzed samples showed high absorbance values at 414 nm, indicating the presence of free hemoglobin [23–25]. Since miR-451 and miR-16 are highly expressed in human hemolyzed blood [25], we also determined their levels in bovine serum. As expected, their expressions were strongly detected in hemolyzed serum compared to non-hemolyzed serum. However, the levels of miR-127, miR-93, and a combination of the two (miR-93/miR-127) were not different in hemolyzed serum samples compared to non-hemolyzed serum samples (Fig 4B). Therefore, we conclude that the combination of miR-93 and miR-127 serves as an adequate reference in bovine serum regardless of hemolysis.

### Validation of candidate reference miRNAs

**Fig 5:**
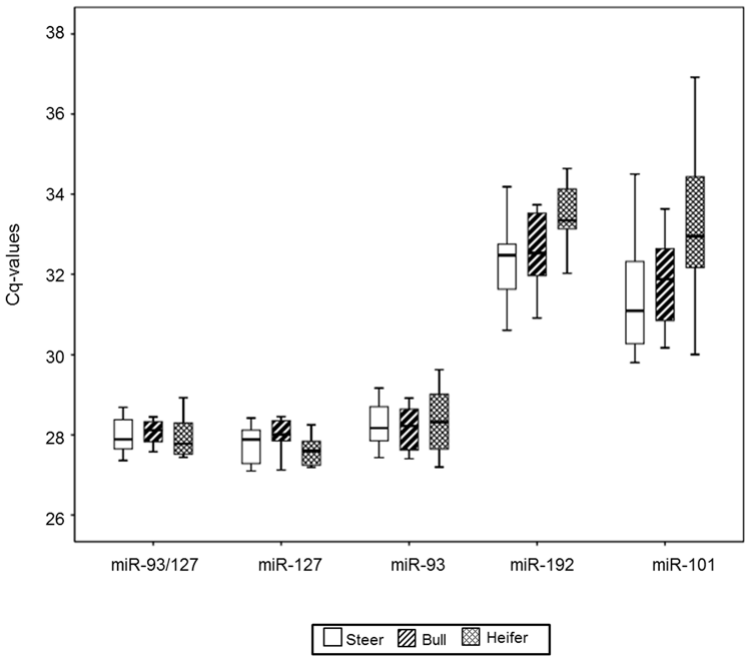
Levels of candidate reference miRNAs among different genders. qRT-PCR experiments were performed on steer serum (steer; n = 14), bull serum (bull; n = 10), and heifer serum (heifer; n = 9). Boxes (blank for steer, diagonal lines for bull, and diamonds for heifer) represent lower and upper quartiles with median.

**Fig 6:**
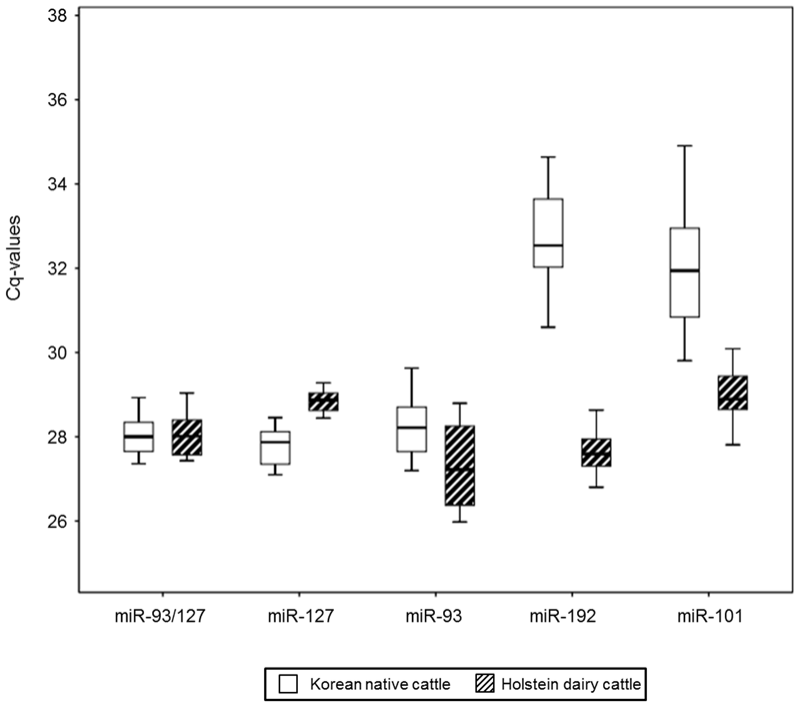
Expression levels of candidate reference miRNAs among different breeds. qRT-PCR analysis was conducted on bovine serum derived from Korean native cattle (n = 34) and Holstein dairy cattle (n = 16). Values are given as real-time PCR cycle threshold numbers (Cq values). Boxes (blank for Korean native cattle and diagonal lines for Holstein dairy cows) represent lower and upper quartiles with median.

To validate further the stability of miR-93, miR-127, and the combination of these two as reference miRNAs, we investigated their expression levels in bovine serum derived from different genders and breeds. First, the levels of candidate miRNAs were analyzed in steer, bull, and heifer serum. miR-93, miR-127, and miR-93/miR-127 were the most highly expressed in all genders, when compared to miR-192 and miR-101 (Fig 5). The Cq value ranges of miR-127 and miR-93/miR-127 were 1.36 and 1.57, respectively. Standard deviation within different gender groups for miR-93, miR-127, and miR-93/miR-127 was less than 1 (S2 Table). Between different gender groups, miR-92/miR-127 showed the least variation, with a p-value of 0.859, the highest value among all the candidate miRNAs. In addition, within different breeds (Holstein dairy cows and Korean native cattle), miR-127 and miR-93/miR-127 had standard deviations of less than 1 (Fig 6 and S3 Table). Only miR-93/miR-127 showed no statistical difference between breeds (p = 0.85). The Cq value range of miR-93/miR-127 in bovine serum extracted from different breeds was the narrowest among the candidate miRNAs. Based on data from different genders and breeds, the combination miR-93/miR-127 is recommended as the optimal reference for miRNAs in bovine serum.

## Discussion

qRT-PCR is a popular tool for accurate miRNA expression profiling [7, 26–28]. Optimal normalization of qRT-PCR data is crucial under different biological conditions [6–7, 29–30]. Circulating miRNAs in body fluid can remain stable not only in the RNase-rich environment of the blood but also in harsh conditions, including boiling temperatures, high or low pH, extended periods of storage, and multiple freeze-thaw cycles [31, 32]. Numerous RNA species, including rRNA, snRNA, and synthetic miRNAs as spike-in controls, have been used as normalizers [26]. However, there is disagreement regarding the abundance and stability of rRNAs and snRNAs in body fluids, and spike-in controls may not correct for the variation arising from differences in template quality and efficiency of the reverse transcription reaction. In addition, miR-191 and miR-103 have been used as endogenous reference miRNAs in human tissues [8]; however, in our study, miR-191 failed to meet the endogenous normalizer criteria, and miR-103 was expressed at a low level in bovine serum. Thus, endogenous miRNAs for solid tissues may not be suitable for bovine serum. In our study, we presented the combination of miR-93/miR-127 as the optimal reference in bovine serum. The expression of this combination was not affected by differences in breed or gender, whereas the individual expressions of miR-93 and miR-127 showed statistical differences within breed groups, indicating that the selection of a single reference miRNA may be not adequate. It is known that a combination of miRNAs is more stable and accurate for normalization than a single reference miRNA [17]. The combination of miR-16 and miR-93 is an adequate reference in human gastric cancer samples [5], and a combination of let-7d, let-7g, and let-7i in human serum is superior to the other single reference genes commonly used for normalization [16]. In mice, miR-146a, miR-16, miR-195, miR-30, and miR-744 have been reported as reference miRNAs in blood [4]. Although previously no study has been carried out for miR-127 and miR-93 in bovine serum, miR-127 is expressed differently between mature and premature oocytes [33], and the level of miR-93 expression depends on the thickness of backfat [34]. These data suggest that miR-93 and miR-127 might not be stably expressed in cattle tissues and are only adequate as reference genes for bovine serum.

Hemolysis can occur in most of the steps carried out during blood sampling and handling procedures. Recent studies have shown that hemolysis occurring during blood collection has a substantial impact on the miRNA content in blood [23–25]. miR-451 and miR-16 exist abundantly in human red blood cells [25]. Since these miRNAs could be affected by disease, tissue or organ damage, and natural variation, they are not suitable as serum reference miRNAs [35]. In our study, the expression levels of miR-451 and miR-16 were higher in hemolyzed serum than in non-hemolyzed serum, whereas the level of miR-93/miR-127 in bovine serum did not vary regardless of hemolysis, indicating that this combination may be optimal as a reference in bovine whole blood cells.

In summary, our results show that the combination miR-93/miR-127 is stably expressed in bovine serum and is an adequate endogenous miRNA reference for RT-qPCR-based detection. This finding could contribute to the evaluation of the levels of miRNAs associated with bovine disease and economic traits as blood metabolites.

## Supporting Information

S1 Table PCR efficiency of candidate reference miRNAs. (DOCX)

S2 Table Cq values of validated reference genes under different gender conditions. (DOCX)

S3 Table Cq values of validated reference genes when evaluated among different breeds. (DOCX)
