# Supplementary material for: Identification of Reference Genes for Relative Quantification of Circulating MicroRNAs in Bovine Serum
Source: PLoS One. 2015 Mar 31;10(3):e0122554. doi: 10.1371/journal.pone.0122554 (PMC4380332; doi:10.1371/journal.pone.0122554)
Supplement: S1 Table — (DOCX) [file pone.0122554.s001.docx]

**Table S1. PCR efficiency of candidate reference miRNAs.**

| Name | PCR efficiency (%) |
| --- | --- |
| let-7a | 99.25 |
| miR-16 | 103.53 |
| miR-23a | 96.84 |
| miR-93 | 98.84 |
| miR-101 | 96.50 |
| miR-127 | 99.25 |
| miR-191 | 96.06 |
| miR-192 | 93.07 |
| miR-195 | 93.80 |
| miR-451 | 96.84 |
