# Supplementary material for: Identification of Reference Genes for Relative Quantification of Circulating MicroRNAs in Bovine Serum
Source: PLoS One. 2015 Mar 31;10(3):e0122554. doi: 10.1371/journal.pone.0122554 (PMC4380332; doi:10.1371/journal.pone.0122554)
Supplement: S2 Table — (DOCX) [file pone.0122554.s002.docx]

**Table S2. Cq values of validated reference genes under different gender conditions.**

| Name | Cq Range | Cq Min | Cq Max | Median Cq ± SD | | | *p* value |
| --- | --- | --- | --- | --- | --- | --- | --- |
|  |  |  |  | Steer | Bull | Heifer |  |
| miR-93/127 | 1.57 | 27.36 | 28.93 | 27.89±0.43 | 28.12±0.30 | 27.78±0.55 | 0.859 |
| miR-127 | 1.36 | 27.1 | 28.46 | 27.89±0.42 | 28.01±0.44 | 27.60±0.38 | 0.147 |
| miR-93 | 2.43 | 27.2 | 29.63 | 28.17±0.56 | 28.22±0.53 | 28.32±0.84 | 0.839 |
| miR-192 | 4.04 | 30.6 | 34.64 | 32.48±1.04 | 32.53±0.96 | 33.35±0.89 | 0.085 |
| miR-101 | 7.11 | 29.81 | 36.92 | 31.09±1.37 | 31.88±1.88 | 32.95±2.10 | 0.070 |
