# Supplementary material for: Identification of Reference Genes for Relative Quantification of Circulating MicroRNAs in Bovine Serum
Source: PLoS One. 2015 Mar 31;10(3):e0122554. doi: 10.1371/journal.pone.0122554 (PMC4380332; doi:10.1371/journal.pone.0122554)
Supplement: S3 Table — (DOCX) [file pone.0122554.s003.docx]

**Table S3. Cq values of validated reference genes when evaluated among different breeds.**

| Name | Cq Range | Cq Min | Cq Max | Median Cq ± SD | | *p* value |
| --- | --- | --- | --- | --- | --- | --- |
|  |  |  |  | Korean native cattle | Holstein dairy cow |  |
| miR-93/127 | 1.68 | 27.36 | 29.03 | 28.00±0.42 | 28.01±0.51 | 0.850 |
| miR-127 | 2.18 | 27.1 | 29.28 | 27.87±0.43 | 28.87±0.26 | *p* < 0.001 |
| miR-93 | 3.65 | 25.98 | 29.63 | 28.22±0.62 | 27.22±1.02 | *p* < 0.001 |
| miR-192 | 7.92 | 26.80 | 34.64 | 32.54±1.06 | 27.59±0.46 | *p* < 0.001 |
| miR-101 | 9.11 | 27.81 | 36.92 | 31.94±1.85 | 28.89±0.68 | *p* < 0.001 |
